# Identification of a Glucose Metabolism-related Signature for prediction of Clinical Prognosis in Clear Cell Renal Cell Carcinoma

**DOI:** 10.7150/jca.45296

**Published:** 2020-06-21

**Authors:** Sheng Wang, Ling Zhang, Zhihong Yu, Kequn Chai, Jiabin Chen

**Affiliations:** 1 The Second Clinical Medical College, Zhejiang Chinese Medicine University, Hangzhou, Zhejiang.; 2 Department of Oncology, Tongde Hospital of Zhejiang, Hangzhou, Zhejiang 310053, P.R. China.

**Keywords:** glucose metabolism, clear cell renal cell carcinoma, signature

## Abstract

**Background:** Clear cell renal cell carcinoma (ccRCC) is one of the most prevalent and invasive histological subtypes among all renal cell carcinomas (RCC). Cancer cell metabolism, particularly glucose metabolism, has been reported as a hallmark of cancer. However, the characteristics of glucose metabolism-related gene sets in ccRCC have not been systematically profiled.

**Methods:** In this study, we downloaded a gene expression profile and glucose metabolism-related gene set from TCGA (The Cancer Genome Altas) and MSigDB, respectively, to analyze the characteristics of glucose metabolism-related gene sets in ccRCC. We used a multivariable Cox regression analysis to develop a risk signature, which divided patients into low- and high- risk groups. In addition, a nomogram that combined the risk signature and clinical characteristics was created for predicting the 3- and 5-year overall survival (OS) of ccRCC. The accuracy of the nomogram prediction was evaluated using the area under the receiver operating characteristic curve (AUC) and a calibration plot.

**Results:** A total of 231 glucose metabolism-related genes were found, and 68 differentially expressed genes (DEGs) were identified. After screening by univariate regression analysis, LASSO regression analysis and multivariable Cox regression analysis, six glucose metabolism-related DEGs (*FBP1, GYG2, KAT2A, LGALS1, PFKP,* and* RGN*) were selected to develop a risk signature. There were significant differences in the clinical features (Fuhrman nuclear grade and TNM stage) between the high- and low-risk groups. The multivariable Cox regression indicated that the risk score was independent of the prognostic factors (training set: HR=3.393, 95% CI [2.025, 5.685], p<0.001; validation set: HR=1.933, 95% CI [1.130, 3.308], p=0.016). The AUCs of the nomograms for the 3-year OS in the training and validation sets were 0.808 and 0.819, respectively, and 0.777 and 0.796, respectively, for the 5- year OS.

**Conclusion:** We demonstrated a novel glucose metabolism-related risk signature for predicting the prognosis of ccRCC. However, additional *in vitro* and *in vivo* research is required to validate our findings.

## Introduction

Renal cell carcinoma (RCC) is one of the most prevalent cancers 1 and was ranked the sixth deadliest malignant tumor worldwide in 2018 2. According to The American Cancer Society, in the USA alone, it was estimated that more than 73,000 new cases of RCC would be diagnosed by the end of 2019 and over 14,000 people would die from it in 2019 3. Clear cell renal cell carcinoma (ccRCC) is the most frequent and invasive histological subtype among all types of RCC and accounts for 70-80% of RCC cases 4,5. However, with modern diagnostic and treatment methods, RCC-related deaths are constantly decreasing 6, and the 5-year survival rate of patients in advanced stages is 10% 7. Moreover, up to 40% of RCC patients have developed metastasis after surgical intervention 8. The high mortality rate of ccRCC patients in advanced stages may result from lack of effective treatments and reliable risk stratification for assessing the prognosis. The TNM classification system and Fuhrman nuclear grade are the most commonly used clinicopathological parameters for clinical decision making and the prognosis stratification of RCC 9. However, an increasing body of literature has reported differences in clinical outcomes among RCC patients with the same TNM stage and similar therapeutic regimens 10,11, suggesting that the TNM staging system alone cannot provide complete information for the prognostication of RCC. Therefore, there is an urgent need to identify tumor-specify biomarkers and develop useful clinical prognostic markers for the precise prediction of outcome, which may contribute to risk stratification and guide the clinical diagnosis and treatment of ccRCC.

In the past 15 years, cancer cell metabolism, particularly glucose metabolism, has attracted many researchers and has been proposed as a hallmark of cancer 12. In the tumor microenvironment (TME), tumor cells are more dependent on glucose metabolism for energy generation. The Warburg effect, a unique glucose metabolism-related metabolic reprogramming, is characterized by a high rate of aerobic glycolysis, which promotes the intake of glucose and the production of lactic acid to produce lactic acid and reduce mitochondrial oxidative phosphorylation (OXPHO). This process is beneficial to the occurrence and development of tumors 13,14. In addition, increased production of lactate acidifies the TME, creating environmental conditions that can boost tumor proliferation, invasion, and migration 15. Glucose metabolism and the Warburg effect not only support the rapid growth of cancer cells but also reduces the dependence of cancer cells on oxygen availability in the TME 13. Hence, regulation of glucose metabolism may be a novel strategy for the treatment of cancer.

In this study, we obtained the gene expression profile from The Cancer Genome Altas (TCGA, version: April 5, 2018, https://cancergenome.nih.gov) and the glucose metabolism related gene set from Molecular Signatures Database v7.0 (MSigDB, http://www.broad.mit.edu/gsea/msigdb/) 16 to perform a systematic and comprehensive analysis of the characteristics of the glucose metabolism-related gene set of ccRCC. Next, we developed a glucose metabolism-related signature for assessing the prognosis of ccRCC patients. In addition, genes highly associated with the risk signature were identified for a functional enrichment analysis and protein-protein interaction (PPI) network. The novel prognostic signature is warranted for the improvement of treatment selection and outcome prediction compared to TNM staging and may help with the development of novel strategies for diagnosis and the identification of potential drug targets of ccRCC.

## Materials and Methods

### Data source and preprocessing

The gene expression profiles, as well as the relevant clinical characteristics, were downloaded from TCGA. Patients with follow‐up times< 30 days or a lack of pathological diagnosis and corresponding clinical information were removed. A total of 515 ccRCC patients (259 cases in the training set and 256 cases in the validation set) were enrolled in this research (Table 1). A glucose metabolism-related gene set was collected from MSigDB. After comparison with the gene expression profile from TCGA, a glucose metabolism-related gene expression profile was identified, which included 231 genes.

### Consensus clustering

The “ConsensusClusterPlus” package of the statistical software R (version 3.5.2, https://www.r-project.org) was used to perform the consensus clustering. The consensus matrices and cumulative distribution function (CDF) were applied for assessing the optimal number of clusters. Principal component analysis (PCA) was carried out to investigate the expression difference between the clusters with the R package “princomp”.

### Construction and evaluation of the prognostic glucose metabolism-related gene signature

The R and “edgeR” Bioconductor packages (http://www.bioconductor.org/packages/release/bioc/html/edgeR.html) were utilized to identify differentially expressed genes (DEGs) with |logFC| > 2 and FDR < 0.01. Patients with ccRCC were divided into two sets (a training set and a validation set) randomly. In the training set, DEGs that were highly associated with the overall survival (OS) of the ccRCC patients were further screened with univariate Cox regression analysis and LASSO regression analysis. Last, a multivariate Cox regression analysis was applied to select the best survival-related candidate DEGs and develop a risk score formula. The following equation was used: Risk score = (coefficient * expression of gene 1) + (coefficient * expression of gene 2) + ... + (coefficient * expression of gene X). With the median value of the risk score as the cut-off value, patients were divided into low-risk and high-risk groups. In the same way, the risk score of each individual in the validation set was also calculated. In addition, the receiver operating characteristic curve (ROC) and the calibration plot with a boot-strapping set of 1,000 resamples was used to evaluate the predictive capacity of the prognostic signature.

### Functional enrichment analysis and protein-protein interaction (PPI) network construction

The Pearson correlation coefficients between candidate risk scores and genes in the expression matrix were calculated. Genes with a correlation coefficient P>0.45 or P<0.01 were selected for further investigation. Kyoto Encyclopedia of Genes and Genomes (KEGG) and Gene Ontology (GO) were performed using DAVID (Database for Annotation, Visualization and Integrated Discovery, version 6.8, https://david-d.ncifcrf.gov) (17) and the PPI network was constructed using String (version 10.5, https://string-db.org) 18.

### Survival Analysis

Kaplan-Meier plots were generated to illustrate the survival relationship between the risk score and the OS of ccRCC patients. Univariable and multivariable Cox regression models were used to determine the independent prognostic factors. A P-value<0.05 was set as the cut-off value.

## Results

### Stratification of ccRCC based on the glucose metabolism-related gene set

**Figure 1:**
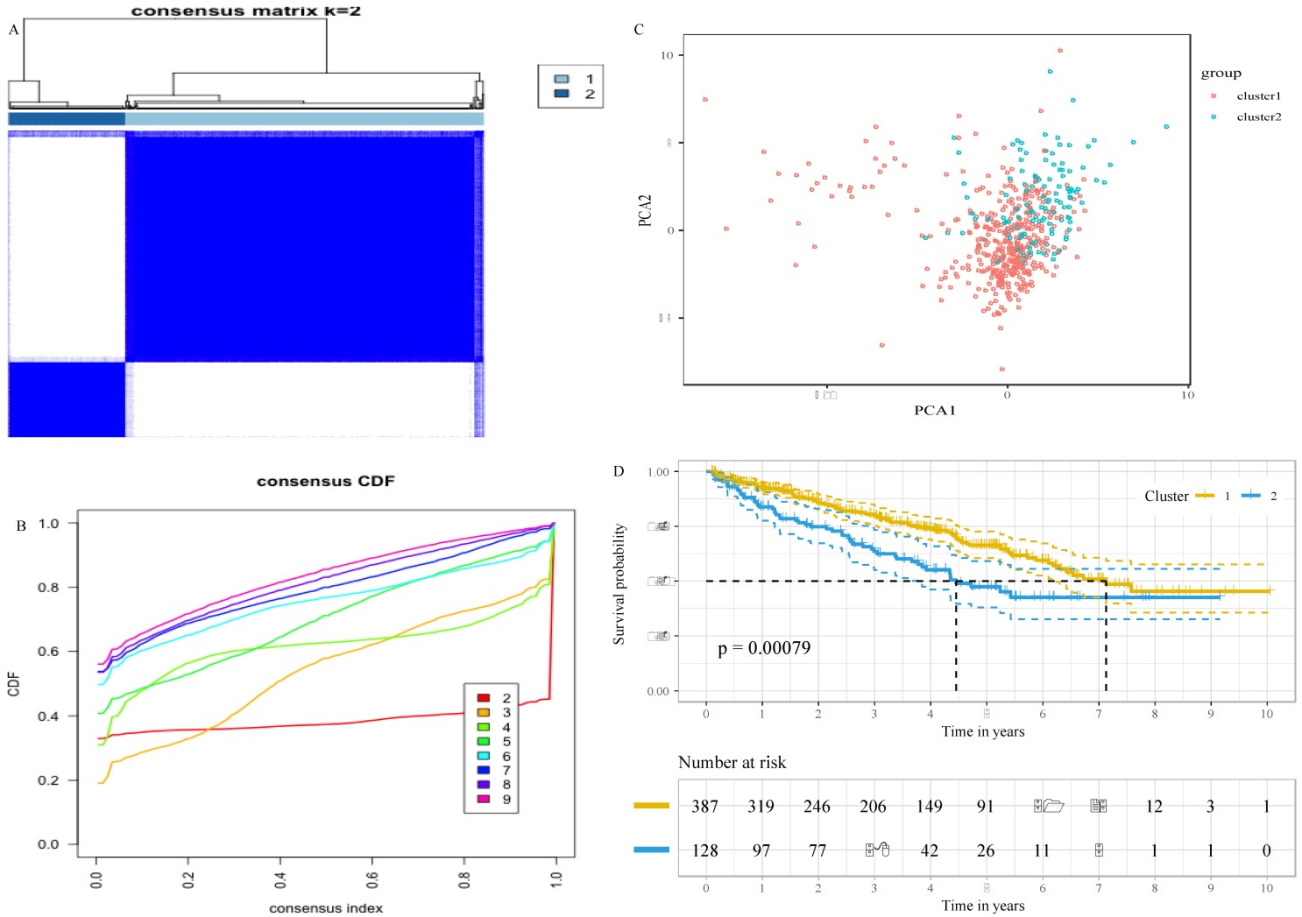
Stratification of ccRCC based on the glucose metabolism-related gene set. A, Consensus clustering matrix of 515 ccRCC samples for k = 2. B, Consensus clustering CDF for k = 2 to k = 10. C, Principal component analysis (PCA) of cluster 1 and cluster 2 based on whole gene expression data. D, survival analysis of ccRCC patients in cluster 1 and cluster 2.

To decipher the relationship between the glucose metabolism-related genes and the outcomes of ccRCC patients, we classified 515 patients into two robust clusters (K=2) with the “ConsensusClusterPlus” package in R (Figure 1A and B). PCA revealed two clusters within different areas, indicating that the differences between the two clusters were greater than those within the cluster (Figure 1C). A Chi-square test revealed that there were significant differences in the TNM stage, grade and survival status between the two clusters (Table 2). In addition, the survival analysis showed that patients in cluster 2 had a worse prognosis compared with patients in cluster 1 (Figure 1D). These results indicate that the expression of glucose metabolism-related genes are highly related with the prognosis and molecular features of ccRCC patients.

### Construction and evaluation of the prognostic glucose metabolism-related gene signature

**Figure 2:**
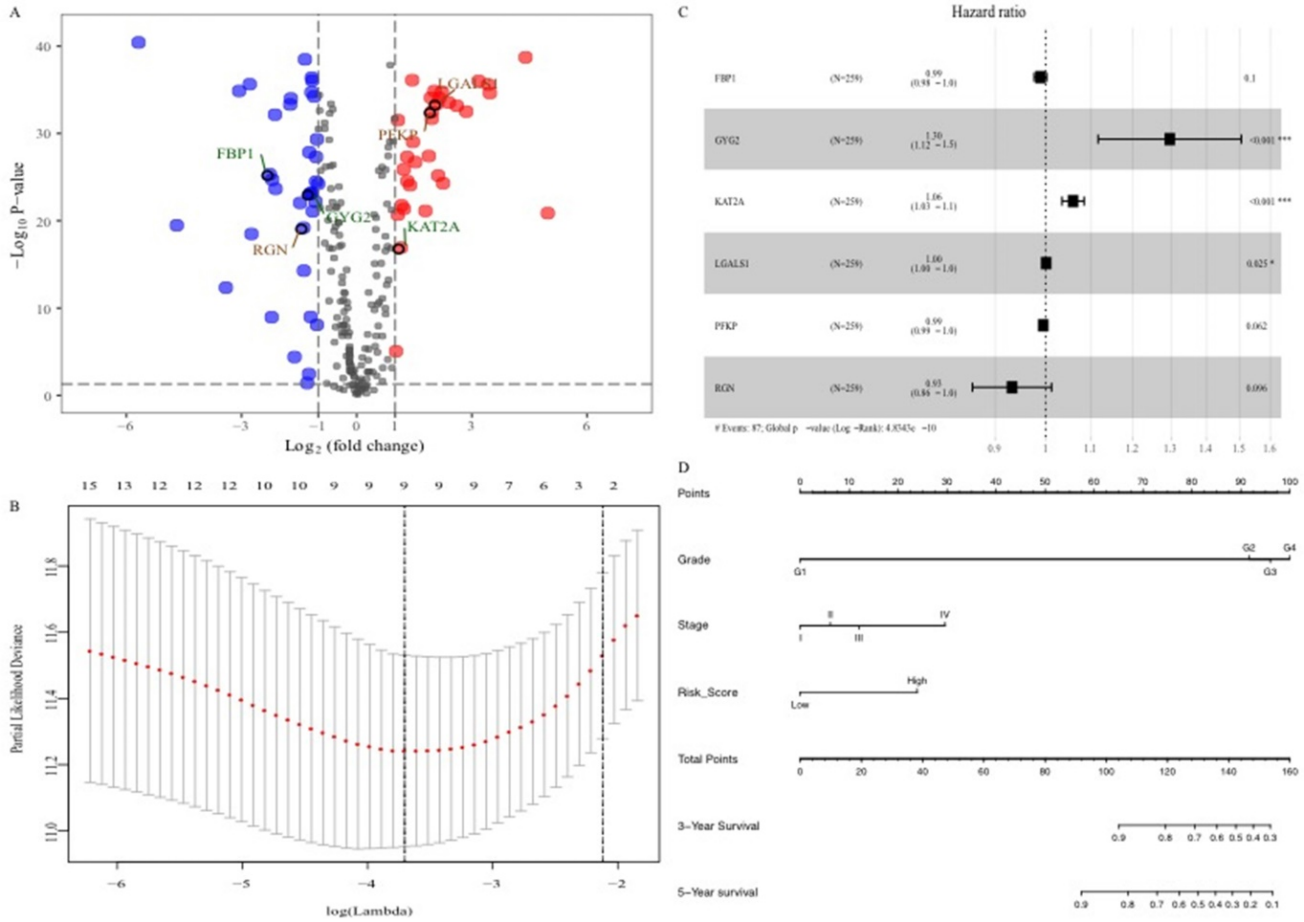
Identification of the six glucose metabolism-related genes signature. A, Volcano plot of DEGs. Red dots represent 32 upregulated genes and green dots represent 36 downregulated genes. B, “Leave- one-out-cross-validation” for parameter selection in LASSO regression. C, The forest map of multivariate Cox regression analysis. D, The prognostic nomogram for the prediction of 3- and 5-year overall survival in ccRCC.

**Figure 3:**
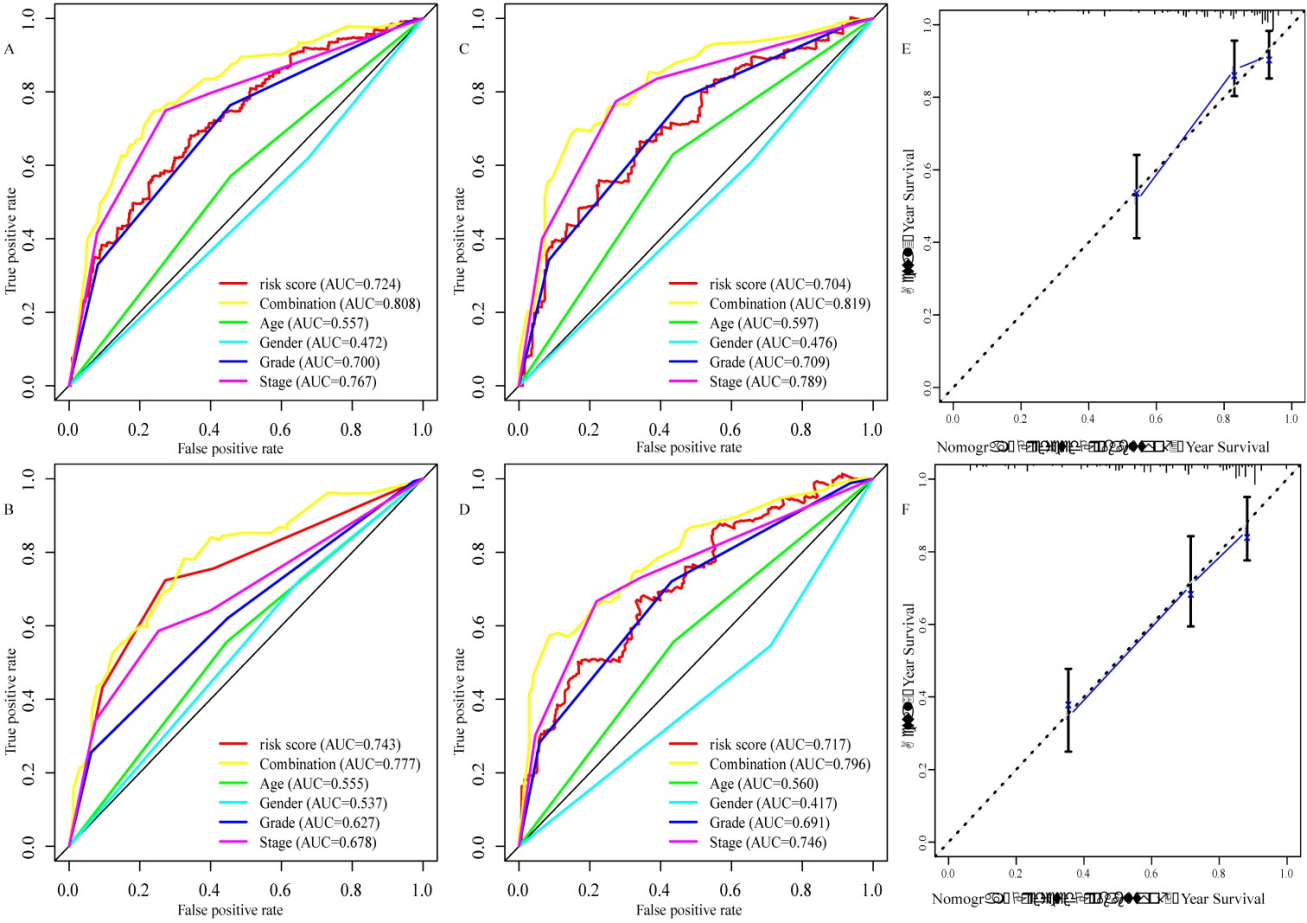
Evaluation of the six glucose metabolism-related genes signature. A, the area under the receiver operating characteristic (ROC) curve (AUC) for the 3-year overall survival of ccRCC patients in the training set. B, the AUC for the 5-year overall survival of ccRCC patients in the training set. C the AUC for 3-year overall survival of the ccRCC patients in the validation set. D, the AUC for 5-year overall survival of the ccRCC patients in the validation set. E, Calibration curve of the nomogram model for the 3- year overall survival in the training set. F, Calibration curve of the nomogram model for the 5- year overall survival in the training set.

To explore the glucose metabolism-related gene signature, we divided the 515 ccRCC patients into a training set (256 cases) and a validation set (255 cases) randomly. Next, we identified 68 glucose metabolism-related DEGs between ccRCC tissues and adjacent nontumor tissues in the training set, including 32 upregulated DEGs and 36 downregulated DEGs (Figure 2A). After performing univariate Cox regression analysis and LASSO regression analysis, 9 DEGs were filtered out and then subjected to multivariate Cox regression (Figure 2B). Finally, the six best survival-related candidate DEGs (*FBP1, GYG2, KAT2A, LGALS1, PFKP,* and* RGN*) were selected to develop a prognostic gene signature and risk score formula (Figure 2C). A nomogram combining the risk score and clinical characteristics was created for predicting the 3-and 5- year survival rates (Figure 2D). The the area under the curve (AUCs) of the nomograms for the 3- and 5-year OS in the training set were 0.808 and 0.777, respectively (Figure 3A and B). To confirm the accuracy of the survival probabilities of this nomogram, 215 ccRCC patients in the validation set were tested to validate the finding in training cohort. The AUCs for predicting the 3- and 5-year OS in the validation set were 0.819 and 0.796, respectively (Figure 3C and D). The calibration plots for the 3- or 5-year survival probabilities in the training set are shown in Figure 3E and F, and those of the validation set are shown in Figure S1. The distribution of the risk scores, survival probabilities and expression profiles of the six candidate DEGs are shown in Figure S2.

### Correlation between risk score and clinical characteristics

**Figure 4:**
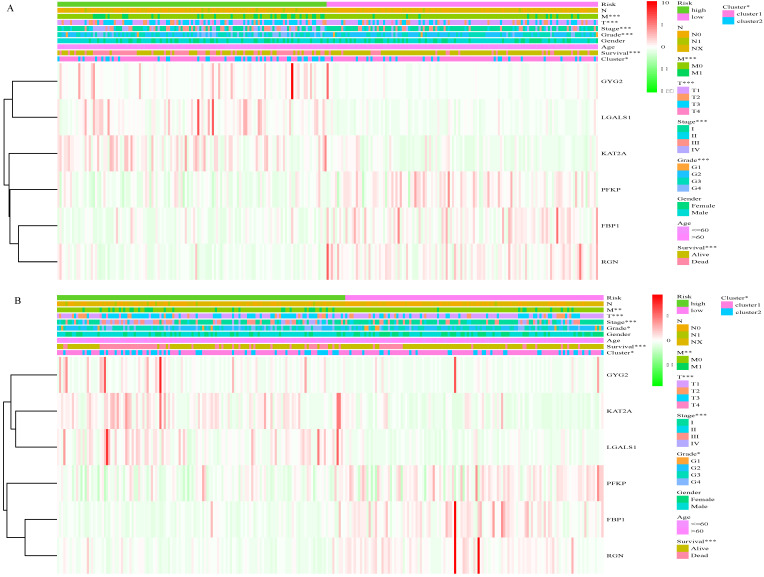
Correlation between risk score and clinical characteristics. A, Heat map of the association of risk scores and clinicopathological features in the training set. B, Heat map of the association of risk scores and clinicopathological features in the validation set. *P<0.05, ** P<0.01, ***P<0.001.

Based on the cut-off values for the risk score (0.840), patients were divided into high- and low- risk groups. As shown in Figure 4A and Table S1, the risk score of each patient was distributed differently in the training set. There were significant differences in most features between the high- and low-risk groups, except gender and age. Similar results were observed in the validation set (Figure 4B, Table S1). This finding indicates a powerful correlation between the glucose metabolism- related signature and the clinical characteristics of ccRCC.

### The six-gene signature is an independent prognostic factor of survival

**Figure 5:**
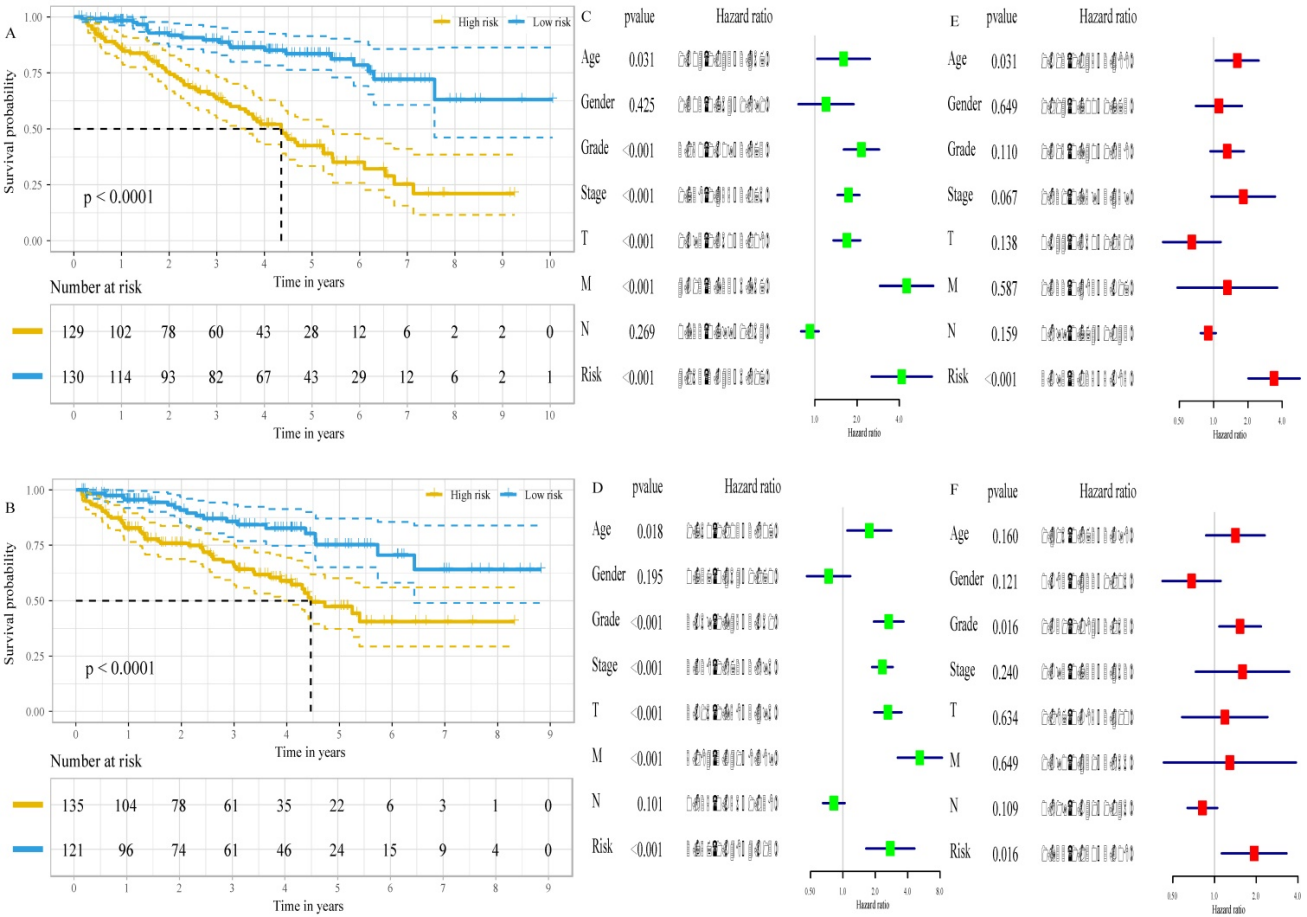
The six-gene signature is an independent prognostic factor of survival. A, Kaplan-Meier survival curves for low- and high- risk groups in the training set. B, Kaplan-Meier survival curves for low- and high- risk groups in the validation set. C, the result of univariable Cox regression analysis in the training set. D, the result of univariable Cox regression analysis in the validation set. E, the result of multivariable Cox regression analysis in the training set. F, the result of multivariable Cox regression analysis in the validation set.

The associations of the risk scores and corresponding overall survival rates were analyzed using a Kaplan-Meier plot and evaluated with a log-rank test. The Kaplan-Meier plot demonstrated that the prognosis of ccRCC patients at high-risk were worse than that at low-risk, both in the training set (P<0.001, Figure 5A) and in the validation set (P<0.001, Figure 5B). Additionally, we found that low expression of *FBP1* (P<0.001, Figure S3A), PFKP (P<0.001, Figure S3B), and RGN (P<0.001, Figure S3C) and high expression of *GYG2* (P<0.001, Figure S3D), *KAT2A* (P<0.001, Figure S3E), and *LGALS1* (P<0.001, Figure S3F) was negatively correlated with a favorable outcome in the ccRCC patients. To compare the risk score with conventional clinical characteristics such as age, gender, TNM stage and grade, we performed univariate and multivariate Cox regression analysis to assess the importance of these indicators for the prognosis of the ccRCC patients. The univariable Cox regression model showed that the risk score was an important factor in the patient prognosis (training set: HR=4.163, 95% CI [2.543, 6.817], p<0.001, Figure 5C; validation set: HR=2.757, 95% CI [1.647, 4.613], p<0.001, Figure 5D). In addition, the results of the multivariable Cox regression indicated that the risk score was independent of the prognostic factors (training set: HR=3.393, 95% CI [2.025, 5.685], p<0.001, Figure 5E; validation set: HR=1.933, 95% CI [1.130, 3.308], p=0.016, Figure 5F).

### Functional enrichment analysis and PPI network

**Figure 6:**
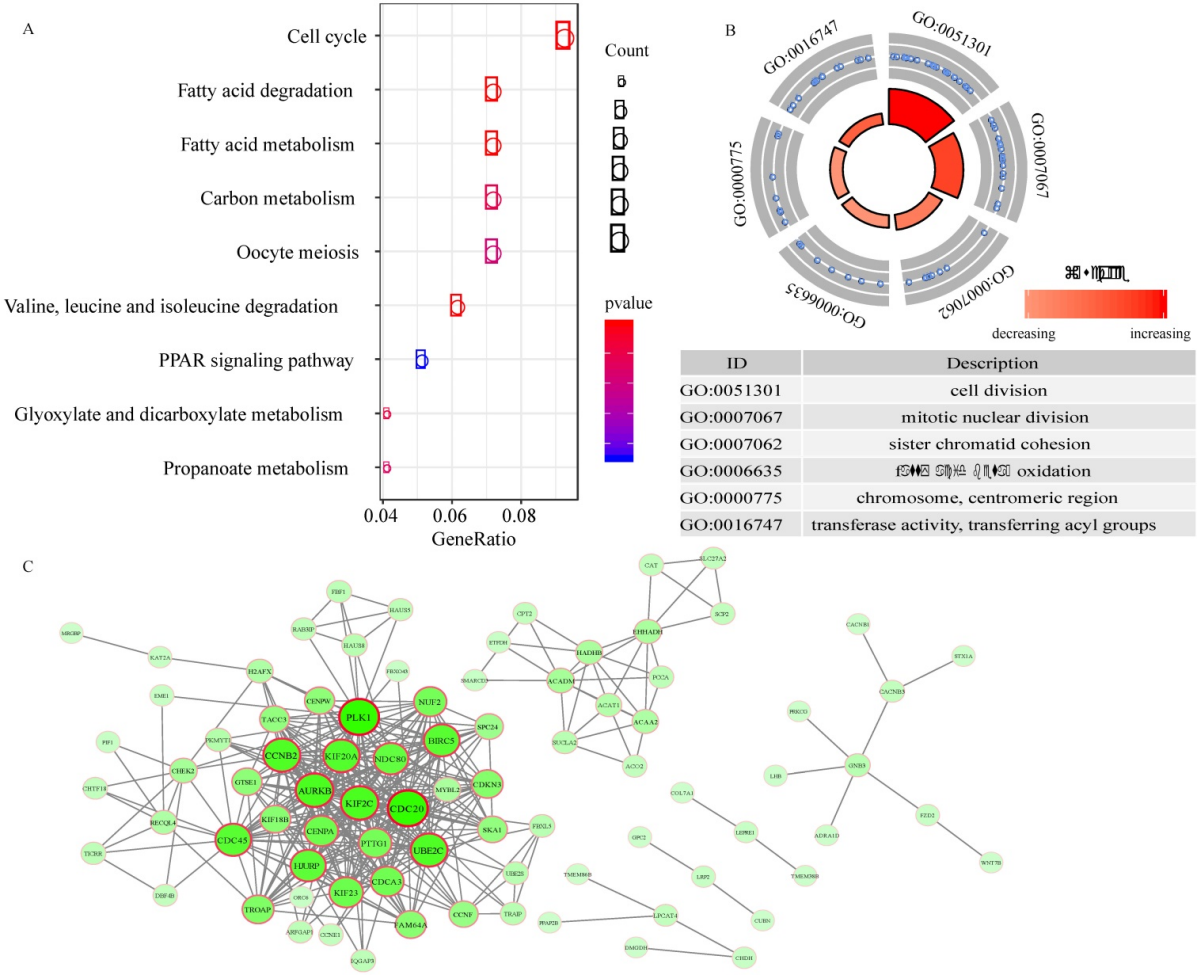
Functional enrichment analysis and PPI network. A, the pathways enriched for 217 genes highly related with risk score. B, GO enrichment analysis. C, Protein-protein interaction (PPI) network.

A total of 217 genes with expression that were highly related to the risk score (Pearson correlation coefficient >0.45 and P<0.01) were identified. The KEGG analysis revealed that these genes were involved in 9 pathways, including the cell cycle, fatty acid degradation, fatty acid metabolism, carbon metabolism, oocyte meiosis, valine, leucine and isoleucine degradation, PPAR signaling pathway, glyoxylate and dicarboxylate metabolism, and propanoate metabolism pathways (Figure 6A). In addition, 6 GO terms (3 biological processes, 1 cellular component, and 1 molecular function) were enriched (Figure 6B).

To explore the interplay among 89 overlapping DEGs, a PPI network was created using the STRING tool with confidence >0.9 as a cut-off criterion. The PPI network contained 71 nodes and 376 edges (Figure 6C).

## Discussion

ccRCC is one of the most prevalent kidney cancers (19) and accounts for approximately 3% of adult malignant tumors 20. The 5-year survival rate of ccRCC patients in advanced stages is less than 10%, and 20-40% patients have experienced distant metastasis at the time of diagnosis 21. The identification of tumor-specific markers and risk stratification is important for assessing the prognosis of patients, which may facilitate the development of new strategies for ccRCC diagnosis and therapy. Moreover, predicting the prognosis is important for treatment selection and the identification of prognosis-related biomarkers 22.

Recently, glucose metabolism was shown to play a critical role in the initiation and progression of various cancers 13. Due to lipid and glycogen accumulation, the change in cytoplasm is the most striking morphological characteristic of ccRCC, which indicates reprogramming glucose metabolism is a crucial factor for the cancerogenesis and progression of ccRCC 23. The down-regulation of the tricarboxylic acid (TCA) cycle and up-regulation of Warburg effect (aerobic glycolysis) is major alteration 23,24. Meanwhile, a study found Warburg effect is a grade-dependent feature and could modulate cell viability and proliferation in ccRCC 25. In addition to these, partition of glycolytic flux can be activated in order to generate the building blocks required for cancer cell growth in ccRCC 26. Previously, transcriptomics and metabolomics revealed the pentose phosphate pathway (PPP) is also up-regulated, which start from glucose-6-phosphate, generates precursors for nucleotide biosynthesis and NADPH for anabolic reactions and redox homeostasis maintenance 27. Research has proved that the rate-limiting enzyme: Glucose-6-phosphate dehydrogenase could promote both anabolic reactions and redox homeostasis 28. Furthermore, Glucose-6-phosphate isomerase is over-expression in ccRCC, and highly associated with the prognosis of ccRCC patients 29. In addition, mitochondrial dysfunction and attenuated mitochondrial respiration chain are also observed in ccRCC, which may bring by the overexpression of *NDUFA4L2* 30. Thus, in this study, we downloaded the gene expression profiles and glucose metabolism-related gene sets from TCGA and MSigDB, respectively, to identify the prognostic glucose metabolism-related gene signatures of ccRCC. Altogether, 68 DEGs (32 upregulated and 36 downregulated) were identified between the ccRCC tissues and adjacent nontumor tissues. After performing univariate regression, LASSO regression and multivariable Cox regression analyses, six glucose metabolism related DEGs (*FBP1, GYG2, KAT2A, LGALS1, PFKP,* and* RGN*) were selected to develop a risk signature for the prediction of ccRCC clinical prognosis. Additionally, survival analysis revealed that all six glucose metabolism related genes were closely correlated with the clinical outcomes of the ccRCC patients. *FBP1* (fructose-bisphosphatase 1), a rate-controlling enzyme in gluconeogenesis, catalyzes the hydrolysis of fructose 1,6-bisphosphate to fructose 6-phosphate 31. Similar to the findings in this study, Ning et al. found that *FBP1* was decreased in ccRCC tissues compared with adjacent healthy tissues 32. In addition, previous studies have shown that high FBP1 expression inhibits tumor growth by hindering epithelial-mesenchymal transition (EMT) 33, and degradation of *FBP1* promotes tumor progression by altering the Warburg effect in hepatocellular carcinoma cells 34. *PFKP* (phosphofructokinase, platelet) is an isoform of phosphofructokinase, which plays a vital role in glycolysis regulation and metabolic reprogramming in many cancers including ccRCC 35,36. *LGALS1* (galectin 1) is a β-galactoside-binding protein that recognizes glycoconjugates and regulates cell proliferation, differentiation and apoptosis in cancer 37. Li et al. showed that *LGALS1* was increased in ccRCC and that high expression of *LGALS1* predicted a poor prognosis 38. *KAT2A* (lysine acetyltransferase 2A) regulates acetyltransferase and succinyltransferase as a transcriptional activator 39. The activation of *KAT2A* can promote nasopharyngeal carcinoma cell proliferation 40 and lung cancer cell apoptosis 41. *RGN* (regucalcin) is preferentially expressed in the kidney and liver and is highly associated with the pentose phosphate pathway 42. A study on zebrafish demonstrated that *RGN* was downregulated in hepatocellular and cholangiocellular carcinomas and played a significant role in cell proliferation and tumorigenesis 42. *GYG2* (glycogenin 2) is a self-glycosylating protein that initiates glycogen biosynthesis and accelerates glucose and galactose metabolism 43.

We created a nomogram that combines risk signature and clinical characteristics to predict the 3- and 5-year OS of ccRCC patients. In addition, we further explored the predictive accuracy of the nomogram for the survival rates at 3- or 5-years. The AUCs of the nomograms for 3- year OS in the training and validation sets was 0.808 and 0.819, respectively, which was significantly higher than those based on clinical characteristics, such as age (training set: 0.557; validation set: 0.579), gender (training set: 0.472; validation set: 0.476), grade (training set: 0.700; validation set: 709) and TNM stage (training set: 0.767; validation set: 0.789). Similar results were observed for the AUC of the nomogram for the 5- year OS (training set: 0.777; validation set: 0.796), and these results were also superior to those based on clinical characteristics (age [training set: 0.555; validation set: 0.560], gender [training set: 0.537; validation set: 0.417], grade [training set: 0.627; validation set: 691] and TNM stage [training set: 0.678; validation set: 0.746]). Recently, a prognostic signature has been used to explore prognosis-related biomarkers and evaluate the prognosis of ccRCC patients. For instance, Chen et al. used 3 mRNAs (*CENPW, FOXM1,* and* NUF2*) to establish a prognostic signature that predicted the 3- and 5- year OS of ccRCC patients 44. In addition, a prognostic signature based on 4 mRNAs (*PTEN, PIK3C2A, ITPA,* and* BCL3*) for predicting the 5- year OS in ccRCC was identified in a study by Dai et al. 45. Luo et al. identified a signature for assessing the 3- and 5- year survival rate in ccRCC patients using 3 miRNAs (miR-130b, miR‐18a, miR‐223) 46. In addition, Shi et al. developed a prognostic signature for predicting the 3-year OS using 5 lncRNAs (ENSG00000229178, ENSG00000236453, ENSG00000245060, ENSG00000258789, and ENSG00000272558) 47. The AUCs of 3- and 5- year OS in a study from Chen et al. were 0.645 and 0.705, respectively, and 0.692 and 0.702, respectively, from Luo et al. The AUCs in Chen's and Luo's studies were smaller compared to those in the training set (0.808 and 0.777) and validation set (0.819 and 0.796) of this study. Furthermore, in Dai's study, the AUC for the 5- year OS was 0.701, which was lower than that of the present study. Moreover, the AUC for 3-year OS in the training cohort and validation cohort of this study (0.808 and 0.819) was preferable to that of Shi et al. (0.703 and 0.630). Additionally, the calibration plot for the 3- or 5-year OS demonstrated consistency between the prediction by the nomogram and the actual observation. All data suggested that the established prognostic nomogram is suitable for estimating the probability of 3- and 5- year overall survival rates of ccRCC patients.

Among different grade and TNM stage, there were obvious differences in the distribution of risk score of patients both in training set and validation set. Moreover, survival analysis for low- and high-risk group indicated that the high-risk group had a poorer prognosis than the low-risk group. Meanwhile, multivariable Cox regression revealed that risk score was independent prognostic factors. These results suggested the glucose metabolism-related signature could serve as a robust indicator in predicting the prognosis of the ccRCC patients and stratify patients for glucose metabolism-targeted therapies in future.

Although the prognostic glucose metabolism-related signature demonstrated a well predictive accuracy for ccRCC patients in this study, there are a few limitations needed to be addressed. Firstly, due to all patients were gathered from public database, the potential of selection bias could not be excluded. Secondly, there was no experimental research conducted to examine the functions of six glucose metabolism-related in ccRCC. Thus further investigation both *in vitro* and *in vivo* is demanded to testify the discovery of this research.

## Conclusion

In this study, we investigated the glucose metabolism-related gene set in ccRCC and its prognostic value and developed a prognostic risk signature based on six glucose metabolism-related genes (*FBP1, GYG2, KAT2A, LGALS1, PFKP,* and* RGN*). By combining a risk signature and clinical information, a prognostic nomogram was created for predicting the 3- and 5- year overall survival, which could contribute to the clinical outcome prediction ability of the TNM staging system and provide a convenient tool for risk assessment. Our findings provide a new understanding of glucose metabolism status and will benefit glucose metabolism-targeted therapies in ccRCC patients. However, additional *in vitro* and *in vivo* research is required.

## Supplementary Material

Supplementary figures and tables.

## Figures and Tables

**Table 1:** Characteristics of ccRCC patients in two sets

Parameter	Training set (n=259)	Validation set (n=256)	P value
**Age**			0.962
≤60	133(51.4%)	132(51.6%)	
>60	126(48.6%)	124(48.4%)	
**Gender**			0.401
Female	83(32.0%)	91(35.5%)	
Male	176(68.0%)	165(64.5%)	
**TNM stage**			0.608
I	132(51.0%)	128(50.0%)	
II	29(11.2%)	26(10.2%)	
III	53(20.4%)	64(25.0%)	
VI	45(17.4%)	38(14.8%)	
**Grade**			0.312
G1	5(2.0%)	11(4.3%)	
G2	121(46.7%)	105(41.0%)	
G3	98(37.8%)	102(39.8%)	
G4	35(13.5%)	38(14.9%)	
**Risk group**			0.506
High	129(49.8)	135(52.7%)	
Low	130(50.2)	121(47.3%)	
**Cluster**			0.194
1	201(77.6%)	186(72.7%)	
2	58(22.4%)	70(27.3%)	
**Survival status**			0.252
Alive	172(66.4%)	182(71.1%)	
Dead	87(33.6%)	74(28.9%)	

**Table 2:** Characteristics of ccRCC patients in cluster 1 and cluster 2

Parameter	Cluster 1 (n=387)	Cluster 2 (n=128)	P value
**Age**			0.559
≤60	202(52.2%)	63(49.2%)	
>60	185(47.8%)	65(50.8%)	
**Gender**			0.054
Female	142(36.7%)	52(40.6%)	
Male	245(63.3%)	76(59.4%)	
**TNM stage**			<0.001
I	215(55.6%)	45(35.2%)	
II	42(10.9%)	13(10.2%)	
III	77(19.9%)	40(31.2%)	
VI	53(13.6%)	30()23.4%	
**Grade**			<0.001
G1	16(4.1%)	0(0.0%)	
G2	185(47.8%)	41(32.0%)	
G3	149(38.5%)	51(39.9)	
G4	37(9.6%)	36(28.1%)	
**Survival status**			0.001
Alive	281(72.6%)	73(57.0%)	
Dead	106(27.4%)	55(43.0%)	

## Abbreviations

RCC: renal cell carcinomas
ccRCC: Clear cell renal cell carcinoma
TCGA: The Cancer Genome Altas
DEGs: differentially expressed genes
OXPHO: mitochondrial oxidative phosphorylation
TME: the tumor microenvironment
CDF: cumulative distribution function
PCA: Principal component analysis
OS: overall survival
ROC: the receiver operating characteristic curve
AUC: the area under the curve
KEGG: Kyoto Encyclopedia of Genes and Genomes
GO: Gene Ontology
PPI: protein-protein interaction
