# Supplementary material for: Identification of a Glucose Metabolism-related Signature for prediction of Clinical Prognosis in Clear Cell Renal Cell Carcinoma
Source: J Cancer. 2020 Jun 21;11(17):4996–5006. doi: 10.7150/jca.45296 (PMC7378912; doi:10.7150/jca.45296)
Supplement: Supplementary file 1 — Supplementary figures and tables. [file jcav11p4996s1.pdf]

Table S1. Correlation risk score and clinicopathological factors of ccRCC patients in the two sets

Parameter	Training set			Validation set		
	High-risk (129)	Low-risk (130)	P value	High-risk (135)	Low-risk (121)	P value
Age			0.757			0.271
≤60	65(50.4%)	68(52.3%)		74(54.8%)	58(47.9%)	
>60	64(49.6%)	62(47.7%)		61(45.2%)	63(52.1%)	
Gender			0.533			0.060
Female	39(30.2%)	44(33.8%)		47(34.8%)	54(44.6%)	
Male	90(69.8%)	86(66.2%)		88(65.2%)	67(55.4%)	
TNM stage			<0.001			0.001
I	51(39.5%)	81(62.3%)		53(39.3%)	75(62.0%)	
II	14(10.9%)	15(11.5%)		13(9.6%)	13(10.7%)	
III	30(23.3%)	23(17.7%)		40(29.6%)	24(19.8%)	
VI	34(26.3%)	11(8.5%)		29(21.5%)	9(7.4%)	
Grade			0.001			0.024
G1	2(1.6%)	3(2.3%)		4(3.0%)	7(5.8%)	
G2	50(38.8%)	71(54.6%)		49(36.3%)	56(46.3%)	
G3	49(38.0%)	49(37.7%)		54(40.0%)	48(39.7%)	
G4	28(21.6%)	7(5.4%)		28(20.7%)	10(8.3%)	
Cluster			0.017			0.047
1	91(70.5%)	110(84.6%)		91(67.4%)	95(78.5%)	
2	38(29.5%)	20(15.4%)		44(32.6%)	26(21.5%)	
Survival status			<0.001			<0.001
Alive	63(48.8%)	109(83.8%)		81(60.0%)	101(83.5%)	
Dead	66(51.7%)	21(16.2%)		54(40.0%)	20(16.5%)	

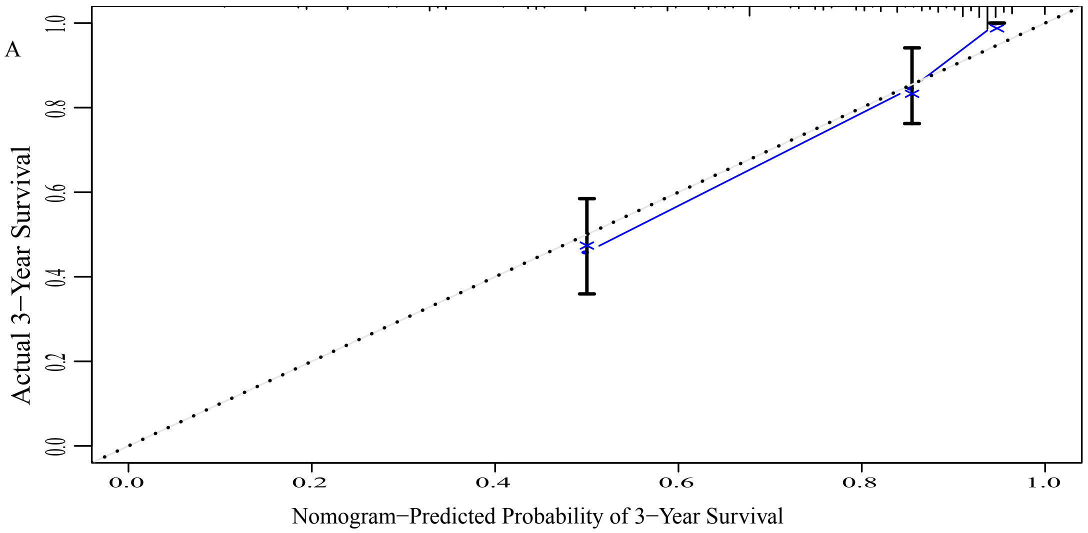

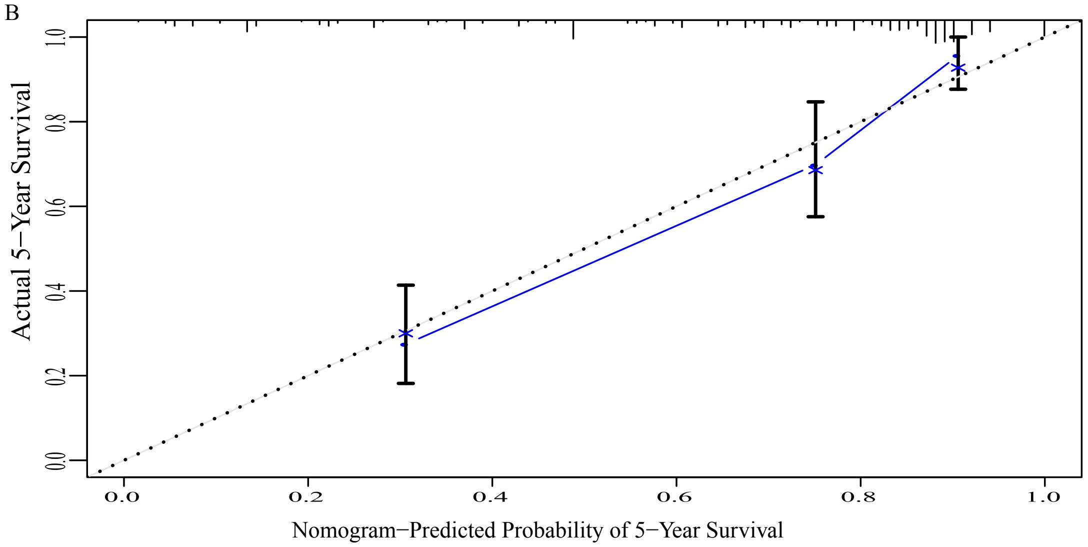

Figure S1. Calibration curve of the nomogram model in validation set. A, for 3- year overall survival. B, for 5- year overall survival.

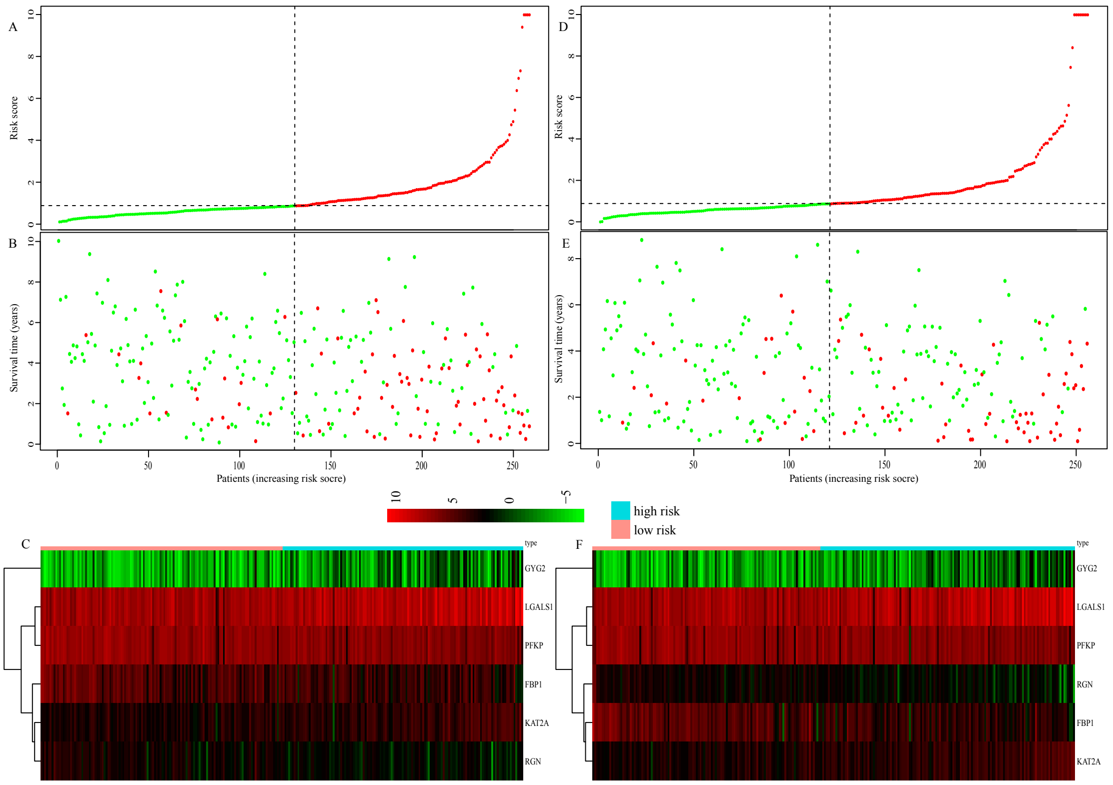

Figure S2. Distribution of risk score, survival statuse and expression profile of 6 six glucose metabolism-related genes. A B and C, in validation set, D E and F, in validation set.

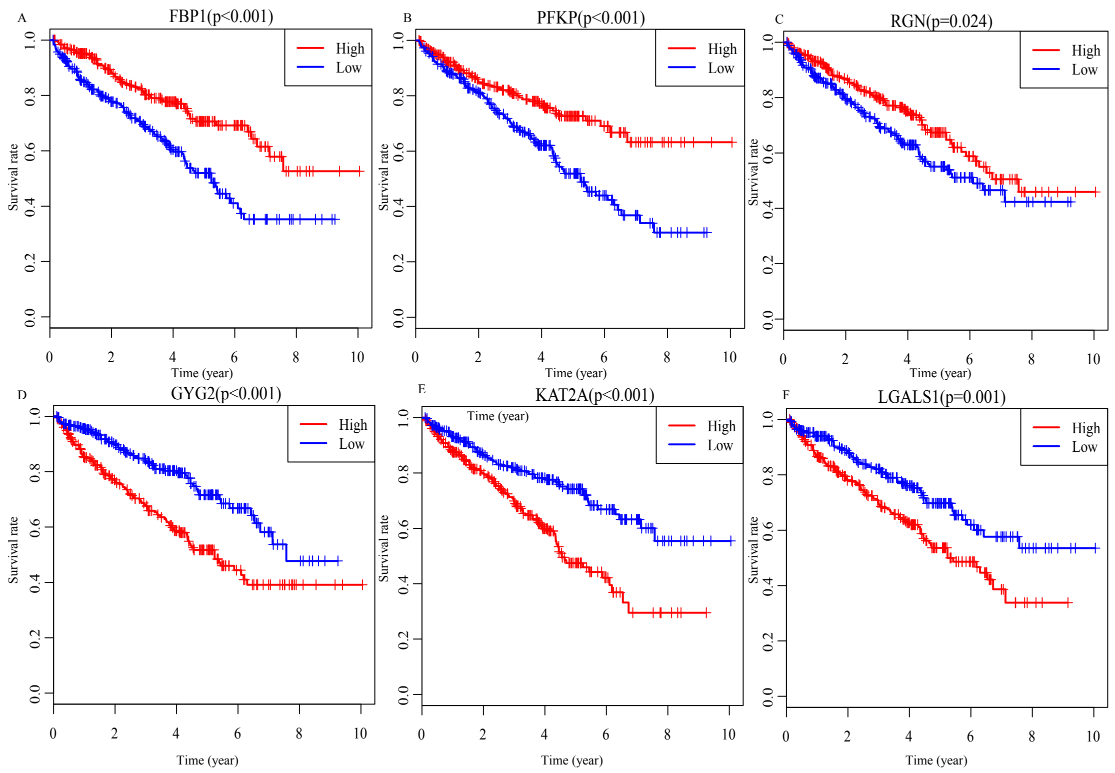

Figure S3. Prognostic values for overall survival. A, FBP1. B, PFKP. C, RGN. D, GYG2. E, KAT2A. F, LGALS1.
